# Identifying and dealing with student errors in the mathematics classroom: Cognitive and motivational requirements

**DOI:** 10.3389/fpsyg.2022.1057730

**Published:** 2022-12-15

**Authors:** Jessica Hoth, Macarena Larrain, Gabriele Kaiser

**Affiliations:** ^1^Institute of Mathematics and Computer Science Education, Faculty of Computer Science and Mathematics, Goethe University Frankfurt, Frankfurt, Germany; ^2^Institute of Mathematics Education, Freiburg University of Education, Freiburg, Germany; ^3^Faculty of Education, University of Hamburg, Hamburg, Germany; ^4^Faculty of Education and Arts, Nord University, Bodø, Norway

**Keywords:** teacher competence, teacher knowledge, teacher beliefs, student errors, teacher noticing

## Abstract

**Introduction:**

Mathematics classrooms are typically characterized by considerable heterogeneity with respect to students’ knowledge and skills. Mathematics teachers need to be highly attentive to students’ thinking, learning difficulties, and any misconceptions that they may develop. Identification of potential errors and appropriate ways to approach them is crucial for attaining positive learning outcomes. This paper explores which knowledge and affective-motivational skills teachers most require to effectively identify and approach students’ errors.

**Methods:**

To address this research question within the German follow-up study of the Teacher Education and Development Study in Mathematics (TEDS-M), 131 primary school mathematics teachers’ ability to identify students’ errors was assessed based on (a) a digitalized speed test showing different students’ solutions in a written notation and (b) three video vignettes that showed different scenes from mathematics classes. These scenes dealt, among other things, with children who struggled with the lesson’s mathematical content. Teachers were asked to analyze students’ thinking and to determine how best to react. In addition, teachers’ mathematics pedagogical content knowledge, mathematical content knowledge, and beliefs were assessed in separate tests and served as predictors for teachers’ abilities to identify, analyze, and deal with students’ errors.

**Results:**

The results indicate that all components are interrelated. However, path analysis reveals that teachers’ ability to deal with students’ errors is mainly predicted by their constructivist beliefs while their ability to quickly identify typical students’ errors is largely dependent on their mathematics content knowledge.

**Discussion:**

The results show the central filtering function of beliefs. Teachers who believe that students must shape and create their own learning processes are more successful in perceiving and analyzing student errors in classroom situations. They may understand errors as learning opportunities and - thus - pay specific attention to these occurrences.

## Introduction

Mathematics learning is a complex process and generally accompanied by instances of irritation and failure. Time and considerations are generally required to overcome these difficulties but may, in turn, facilitate a more profound understanding of the mathematical content.

Mathematical learning processes in school are usually accompanied or even led by teachers, who generally seek to avoid or clarify errors early in the teaching and learning process. However, numerous challenges and influencing factors can impact the effective use of errors in the mathematics classroom; among the other skills required, teachers must be capable of identifying students’ errors and misconceptions. Only if teachers are aware of the incorrect approaches that their students use in class will they be able to reframe errors as learning opportunities. This is closely connected to their understanding and interpretation of what went wrong during teaching and learning and what factors may have caused these misconceptions. Finally, having identified and analyzed the incorrect approach(es), teachers must decide on an effective teaching strategy that will allow their students to develop a more profound understanding and thereby select the most appropriate approach.

During the lesson-planning stage, teachers can decide how much freedom they will allow their students in finding their own approaches to the mathematical content. Strongly teacher-guided lessons offer students fewer opportunities to encounter irritation and failure than more open approaches. However, even if the teacher decides on a teacher-guided discussion, instances of irritation and failure will nonetheless ensue, albeit to a significantly lesser extent. In such instances, teachers can decide how best to proceed. The possibilities for addressing student errors and misconceptions are manifold and dependent on the teachers’ knowledge and the nature of the tasks at hand (Chick and Baker, 2005). Moreover, teachers’ reactions to students’ errors may vary widely, from general avoidance to an appreciation of the potential that errors present as effective learning opportunities. When errors are dismissed outright as undesirable and regarded as a false and unproductive way of student thinking, teachers may decide to simply repeat their explanations or to ignore the student and their mistake, leading to a negative error climate. By contrast, in acknowledging and addressing errors, teachers can create a more positive error climate, in which errors may be used as excellent learning opportunities. Errors may be incorporated into the class discourse and used productively to allow learners to reach a deeper understanding of the mathematical contents (Ingram et al., 2015; Zhao et al., 2018). Empirical findings indicate that error management training and a positive error climate can lead to better learning outcomes than any error prevention strategy (e.g., Keith and Frese, 2008). However, learning from errors is not an automatic process—the teacher plays a central role (Wuttke and Seifried, 2017). Zhao et al. (2018) have empirically demonstrated that the extent to which students learn from their errors is directly affected by the support they receive from their teachers, while teachers’ negative reactions to students’ errors negatively impact students’ affect-motivational adaptability, which, in turn, affects their ability to learn from errors.

Teachers need several abilities to effectively deal with students’ errors and reframe their mistakes as valuable for future learning processes. Specifically, the abilities to identify student errors, to talk about them, and to find strategies that allow to productively work with them in class are regarded as crucial in this context (van Dyk et al., 2005; Stollfuß et al., 2012).

Seifried and Wuttke (2010) identify three facets of competence that teachers need to productively work with students’ errors: (1) content knowledge about the correct solution and possible causes of the error, (2) effective strategies to deal with these errors, and (3) beliefs about the learning potential that errors hold for mathematics education (see also Wuttke and Seifried, 2017). Wuttke and Seifried (2017) explored this aspect as specific to teachers’ competence and called it the “professional error competence of teachers.” Seifried and Wuttke (2017, p. 16) point out that besides “being error-friendly, teachers need to diagnose errors and to identify their potential causes, as well as be able to use them constructively (in the sense of creating opportunities to learn) in the classroom.” Here, teachers’ abilities, such as perception, interpretation, and decision-making skills, become particularly relevant.

In the following section, we define the construct of professional (error) competence and subsequently focus on those components that are considered relevant for dealing with students’ errors and misconceptions.

## Teachers’ professional (error) competence

Teachers’ professional competence is a complex construct and has been the focus of several large-scale research approaches in the last decade. Educational research has advanced a range of different conceptualizations of teachers’ professional competences. Kaiser et al. (2017) differentiated these approaches as either cognitive or situated approaches. Cognitive approaches—as identified by prominent studies, such as the international study TEDS-M (Teacher Education and Development Study in Mathematics, Blömeke and Kaiser, 2014), the COACTIV study (Cognitive Activation in the Classroom, Kunter et al., 2013), or the MTLT project (Mathematics Learning and Learning to Teach Project, Ball et al., 2008)—have invested considerable effort in conceptualizing teachers’ professional competences. These studies regard teachers’ professional competences as including knowledge and affective-motivational aspects, both of which are acknowledged as relevant for successful actions in the teaching profession (Weinert, 2001).

Situated approaches model teachers’ professional competences by considering more situation-specific aspects of the profession. Various approaches are available to conceptualize those competence facets that become relevant during the course of teaching. Kaiser et al. (2015) drew on research in the field of teachers’ expertise and teacher noticing and recognized situation-specific skills as crucial for successful professional teaching. Türling et al. (2012) and Gewiese et al. (2011) provided examples of a situated approach, using video vignettes to assess teachers’ understanding and knowledge of strategies for dealing with students’ errors and difficulties as more situation-specific facets of teachers’ competence. Several other studies have assessed teachers’ competence facets in more situated approaches using video vignettes (Lindmeier et al., 2013; Dunekacke et al., 2015; Bruckmaier et al., 2016; Lazarevic, 2017; Griful-Freixenet et al., 2020; Dreher et al., 2021; Keppens et al., 2021; Larrain and Kaiser, 2022), classroom comic scenes (Herbst et al., 2011; Friesen and Kuntze, 2020), or teaching observations (Schlesinger and Jentsch, 2016).

In their model, which posits competence as a continuum, Blömeke et al. (2015a) suggested that cognitive and situational facets be synthesized in a single model. In this model, competence is understood as a continuum from the disposition traits (knowledge and affective-motivational facets) that underlie the so-called situation-specific skills (perception, interpretation, and decision-making), which mediate the observed behavior in situations of teaching and learning (in the context of teachers’ profession). This understanding of competence as a continuum underpins the conceptualizations of teacher competence in the TEDS-Follow-Up study, to which we refer herein. More precisely, we focus on teachers’ situation-specific skills for dealing with students’ errors and misconceptions. In teaching and learning contexts, teachers must be able to identify students’ errors and misconceptions and analyze them to determine what went wrong in the child’s learning process. Only if mistakes are identified and their causes understood can teachers satisfactorily decide on how further learning should be approached (from errors or in spite of errors). Regarding the model of competence as a continuum, these skills are dependent on teachers’ knowledge and affective-motivational skills. We shall examine each of these facets in greater detail in the sections that follow.

### Teachers’ professional knowledge

Teachers’ knowledge is generally distinguished following the work of Shulman (1986, 1987) in three facets: Content knowledge, pedagogical content knowledge, and general pedagogical knowledge. Content knowledge is specified as knowledge about the subject’s content. In this regard, Shulman (1986, p. 9) clarifies [that] “We expect the subject matter content understanding of the teacher be at least equal to that of his or her lay colleague, the mere subject matter major.” In this regard, mathematics teachers’ content knowledge concerns knowledge about mathematics in general, regardless of the school context. More precisely—and referring to Shulman (1986, ibid.)—mathematics content knowledge is knowledge about the structure of mathematics.

Shulman (1986, p. 9) defines the second facet of teachers’ professional knowledge—pedagogical content knowledge—as “a second kind of content knowledge […], which goes beyond content knowledge of the subject matter per se to the dimension of subject matter knowledge for teaching.” Shulman provides several examples for this specific knowledge facet, such as knowledge about different forms of representations, knowledge about characteristics that hinder or facilitate students’ learning, or knowledge about students’ misconceptions. Finally, the third facet of teachers’ professional knowledge—teachers’ general pedagogical knowledge—is not content-specific.

These knowledge facets are considered central to teachers’ disposition in combination with affective-motivational skills (Blömeke et al., 2015a). While teachers require knowledge, they must also be willing and motivated to cope with problematic incidents that occur in the teaching context. These affective-motivational skills are often operationalized as teachers’ professional beliefs, self-regulation, and motivation (e.g., Blömeke and Delaney, 2014).

Regarding teachers’ professional error competence, Wuttke and Seifried (2017) specify several knowledge facets that have become relevant in dealing with student errors in the context of teaching and learning. Türling et al. (2012) suggested content knowledge—namely, the knowledge of potential error types—as a prerequisite for identifying and productively dealing with student errors. Furthermore, pedagogical content knowledge (PCK) is relevant for adequate feedback as well as for anticipating instructional approaches that promote learning from errors (Wuttke and Seifried, 2017). However, they also declare that “despite rather broad research in this domain, it is still quite uncertain as to exactly what competences teachers should have in order to deal with errors constructively” (Türling et al., 2012, pp. 95–96).

### Teachers’ beliefs

Affective-motivational skills are another central component that is relevant for professional action (Blömeke et al., 2015a). Teachers’ beliefs are considered a crucial facet within the affective-motivational domain (Blömeke and Kaiser, 2017) as they may influence teachers’ instructional practices. Beliefs are defined as “psychologically held understandings, premises, or propositions about the world that are felt to be true” (Richardson, 1996, p. 103). Compared to knowledge, beliefs are more subjective assertions that individuals accept as true but that cannot be externally confirmed or verified and may be resistant to change, even when confronted with evidence that contradicts them (Buehl and Alexander, 2009). In contrast to attitudes and emotions, beliefs include a cognitive component complementing affective aspects and are harder to change (Philipp, 2007). According to Thompson (1992), people may hold different (even contrary) beliefs that are interconnected in a complex belief system. Moreover, beliefs are generally assumed to be context-specific (Pajares, 1992). Given their importance in each individual’s interpretation of their world, “beliefs might be thought of as lenses that affect one’s view of some aspect of the world or as dispositions toward action” (Philipp, 2007, p. 259). Teacher beliefs are thus crucial to the ways in which teachers perceive classroom situations and the instructional decisions they make (Leder et al., 2002). Beliefs are regarded as connecting teachers’ knowledge and action in class (Felbrich et al., 2014). Moreover, two teachers with similar knowledge levels may teach mathematics very differently using different approaches as a result of their divergent beliefs about the nature of mathematics and the possible adequate approaches to teaching and learning in the subject (Ernest, 1989).

Regarding mathematics education, three prominent facets of teachers’ beliefs may be differentiated: beliefs about the nature of mathematics (Grigutsch et al., 1998), beliefs about the teaching of mathematics (Peterson et al., 1989), and beliefs about the learning of mathematics (Heyder et al., 2020). These beliefs can be distinguished into two dimensions: one more traditional and the other constructivist (Blömeke and Kaiser, 2014). The former is characterized by a static perspective on mathematics as a set of rules and procedures and a teacher-centered and transmission-oriented view of mathematics learning. The latter espouses a dynamic view of mathematics as an inquiry-based process and a constructivist view of the learning of mathematics, focusing on students as active participants in their own learning process. Teachers espousing the latter view highlight the relevance of promoting students’ active participation, providing opportunities for students to develop their own strategies for solving problems, facilitating student inquiry, and focusing on the development of mathematical thinking processes.

The central role that beliefs play in the context of teaching and learning mathematics has several empirical indications (e.g., Stipek et al., 2001; Dubberke et al., 2008; Heyder et al., 2020). In addition—and more specifically—teachers’ beliefs are regarded as a central influential facet in recognizing and capitalizing on the benefits of students’ errors (e.g., Seifried and Wuttke, 2010; Türling et al., 2012): “In order to facilitate learning from errors, teachers should believe that errors can be learning opportunities and are not obstacles in the learning process” (Wuttke and Seifried, 2017, p. 5). More precisely, a constructivist approach toward mathematics teaching and learning is expected to accompany a positive error climate and productive learning processes that originate from student errors (Wuttke and Seifried, 2017).

### Situation-specific skills: Perception, interpretation, and decision-making skills

In specific professional teaching and learning situations, teachers require specialized skills. As proposed by situational approaches to teacher competence as well as in reference to work in the fields of teacher noticing (Sherin et al., 2011) and teachers’ expertise (Li and Kaiser, 2011), these are the so-called Perception, Interpretation, and Decision-making skills (PID model, Kaiser et al., 2015). During class, teachers must perceive incidents that are relevant to their teaching and learning. As such, they must pay attention to and identify particular aspects and events that may be important and distinguish them from irrelevant ones. To do this, teachers must also interpret these events so that they can understand them. Teachers can only react to those incidents if they have (a) perceived them and (b) fully understood them. Therefore, these skills are interconnected. After perceiving incidents of interest, teachers must apply their professional knowledge to analyze what happened, what might have caused a particular scene, and what broader principles of teaching and learning are being called into play (Van Es and Sherin, 2002; Van Es and Sherin, 2008). Teachers require this understanding to help them determine how they should react and/or how to proceed in the classroom.

Regarding professional error competence, teachers must initially identify students’ errors, irritations, or misconceptions (perceiving). After identifying a problematic situation or a recognizing that a student is experiencing learning difficulties, they must analyze the phenomenon and the reasons for its occurrence (interpretation). Finally, teachers must decide how to respond to the situation and how to proceed with their class (decision-making). In their conceptualization of teachers’ professional error competence, Seifried and Wuttke (2010, p. 147) specify several points that closely refer to these situation-specific skills: “understanding […] common learner errors and difficulties in learning” (interpretation) and “strategies for dealing with those errors and difficulties” (decision-making). In German-language discourse, in particular, the identification and analysis of student errors and their possible causes are often labeled “diagnostic competence” (e.g., Wuttke and Seifried, 2013; Hoth et al., 2016a; Heinrichs and Kaiser, 2018; Larrain and Kaiser, 2022; Leuders et al., 2022). Research in this field has already indicated that teachers’ diagnostic competence depends on their professional knowledge (Hoth et al., 2016a) and their beliefs (Larrain and Kaiser, 2022) and can be promoted through targeted coaching and learning opportunities (Heinrichs, 2015; Larrain, 2021).

### Interrelations between mathematics teachers’ knowledge, beliefs, and situation-specific skills

As Blömeke et al. (2015a) proposed in their model of competence as a continuum, several components of teachers’ dispositions and situation-specific skills interact and influence teachers’ professional behavior in the context of teaching and learning. Research has provided insight into these relationships (e.g., Wilkins, 2008; Holm and Kajander, 2012; Campbell et al., 2014; König et al., 2014; Dunekacke et al., 2015; Hoth et al., 2016a) and indicated that teachers’ content-specific knowledge (MCK and MPCK) is directly related to students’ mathematics achievements (Campbell et al., 2014). In addition, several approaches find empirical indications in relation to significant connections between mathematics teachers’ beliefs and their content-specific knowledge (Wilkins, 2008; Holm and Kajander, 2012; Campbell et al., 2014). In a path model that was developed with close reference to Ernest (1989) hypothesized model of teachers’ knowledge, beliefs, attitude, and their practice, Wilkins (2008) proposed that teachers’ beliefs about inquiry learning mediate between MCK and teachers’ attitudes and practice. Consistent with the model by Ernest (1989) Wilkins argued that knowledge, beliefs, and attitudes are all relevant for instructional practice. Most importantly, according to Wilkins’ model, teachers’ beliefs in the effectiveness of inquiry-based learning methods exerted the strongest effect on teachers’ practices. This is in line with findings from other approaches. Ball (1991) suggests that mathematics content knowledge is crucial for mathematics teaching. However, Ball points out that the instructional practices of teachers whose knowledge bases are comparable will not be the same owing to their divergent beliefs about mathematics teaching and learning. With her results, Ball provides additional indication about the interconnectedness of the different facets of teachers’ competence: “What each of these teachers does is a function of the interactions among these understandings, assumptions and beliefs” (Ball, 1991, p. 50).

Incorporating situation-specific skills into their analyses, Dunekacke et al. (2015) found that preschool teachers’ abilities to perceive mathematical learning situations and plan appropriate actions can be predicted by their pedagogical content knowledge and beliefs about application orientation. Interesting interconnections between teachers’ knowledge, beliefs, and situation-specific skills, which Dunekacke et al. regarded as indicators of teachers’ performance, are emphasized.

Connecting mathematics teachers’ beliefs with their situation-specific skills and instructional practices, Griful-Freixenet et al. (2020) provide indications that situation-specific skills may not mediate between teachers’ beliefs and their instructional practices. However, they find connections between teachers’ beliefs and their situation-specific skills: “Teachers who believe that their students’ intelligence is more malleable than fixed, have a higher ability to notice significant features of inclusive instructions in video clips” (Griful-Freixenet et al., 2020, p. 11). Findings from other studies support these connections. Keppens et al. (2021) found that teachers with strong constructivist beliefs are better at noticing aspects of inclusive classroom characteristics relating to differentiated instruction. In addition, Roose et al. (2019) found that teachers’ beliefs about diversity and the differentiated curriculum serve as filters for their situation-specific skills. Both studies focus on inclusive classroom situations and reported some highly specific findings focusing on diversity-related aspects of mathematics teaching and learning that also include students with difficulties in learning mathematics, student errors, and misconceptions. The results indicated that constructivist beliefs significantly impact teachers’ situation-specific abilities, meaning that teachers who believe that students construct mathematical meaning themselves through active and autonomous learning more precisely perceive and analyze student’s errors and misconceptions and find more appropriate ways to address these difficulties. Teachers with more constructivist beliefs may also regard errors as natural occurrences in the process of constructing understanding of mathematical relations. In recognizing errors as opportunities for learning, they may intentionally remain alert to errors so that they may repurpose them constructively. Findings from existing research support these connections. However, most focused on the entire field of inclusive classrooms (including high-achieving students; e.g., Roose et al., 2019; Keppens et al., 2021), preschool teachers (Dunekacke et al., 2015), or secondary school teachers (Griful-Freixenet et al., 2020), while others do not focus on situation-specific skills (e.g., Wilkins, 2008). Here, we aim to specify these connections for practicing elementary school teachers in the context of students’ errors and misconceptions.

The research approaches described above all acknowledge the important role that beliefs play in the context of teaching and learning. Knowledge is inarguably a prerequisite for planning and interacting with mathematics in class, but beliefs play a central role for the way in which the mathematics content is represented for the students and how learning is organized in class. Some approaches (e.g., Wilkins, 2008) model beliefs as a mediator between knowledge and teachers’ behavior, while other models (e.g., Dunekacke et al., 2015; Blömeke et al., 2015a) conceptualize knowledge and beliefs as two parallel facets of teachers’ dispositions. As the TEDS-Follow-Up study closely refers to the model of competence as a continuum, we choose an approach to model content-specific knowledge and constructivist beliefs at the same level (as two facets of teachers’ dispositions). In addition, we specify teachers’ abilities to perceive, analyze, and decide on how to deal with students’ errors and misconceptions as a key component in the mathematics classroom.

## Research objectives

As described above, teachers’ professional competence allows them to master various challenges in the context of teaching and learning mathematics, including error management. Like professional competence in general, their professional error competence is based on their professional knowledge (about errors and mathematical learning processes) and affective-motivational skills (such as beliefs about the effectiveness of a positive error climate or—more generally—the nature of mathematics teaching and learning). These disposition facets are activated and integrated by their situation-specific skills into their performance in the classroom. Teachers must identify difficulties that students encounter in their learning, analyze errors and causes of misconceptions, and, finally, decide how to react to those errors.

Specifically, in relation to teachers’ competence in error situations, Seifried and Wuttke (2010, p. 150) identify three facets of professional error competence: (1) knowledge of possible error types, (2) available strategies of action/teacher reaction, and (3) a constructive view of errors and their use in classroom processes. Considering the relevance of knowledge and beliefs as disposition facets and situation-specific skills as mediating between disposition and performance, we wish to examine and offer empirical insight into how these different facets are interconnected as they pertain to the specific issue of dealing with errors and misconceptions in mathematics education. In this regard, we focus on two outcome variables that we use to operationalize teachers’ situation-specific error skills:

- Teachers’ ability to quickly identify students’ errors in written student work (perception)
- Teachers’ ability to analyze students’ errors and misconceptions in classroom situations (interpretation)

Based on the distinction of the three facets of professional error competence, we consider three disposition facets and analyze their influence to be predicting variables:

1. Teachers’ mathematical content knowledge
2. Teachers’ mathematical pedagogical content knowledge
3. Teachers’ constructivist beliefs about the teaching and learning of mathematics

Based on findings from earlier studies—many of which indicate a close connection between constructivist beliefs, pedagogical content-knowledge, and situation-specific skills (e.g., Dunekacke et al., 2015; Roose et al., 2019; Griful-Freixenet et al., 2020; Keppens et al., 2021) as well as those highlighting the relevance of content knowledge for identifying students’ errors (e.g., Shulman, 1986; Ball, 1991;) —and the theoretically defined connections (e.g., Blömeke et al., 2015a), we hypothesize the following interrelations:

*H1*: Teachers’ situation-specific skills to identify typical errors in students’ written work depend significantly on their mathematics content knowledge.

*H2*: Teachers’ situation-specific skills to analyze students’ errors and misconceptions in classroom situations depend significantly on their mathematics pedagogical content knowledge and constructivist beliefs.

## Design of the study and database

To empirically test the hypothesized relations, a secondary analysis was conducted using data from the German TEDS Follow-Up study (TEDS-FU), which is a longitudinal follow-up of the international Teacher Education and Development Study in Mathematics (TEDS-M, Blömeke and Kaiser, 2014). TEDS-M was conducted to internationally compare mathematics teachers’ professional competences. Preservice teachers from 17 countries participated in the assessments. The paper and pencil tests comprised three different parts: (a) a test assessing teachers’ mathematical content knowledge, (b) a test assessing teachers’ pedagogical content knowledge, and (c) various questionnaires assessing different aspects, such as learning opportunities, beliefs about the nature of mathematics, beliefs about learning and instruction, and several other personal and institutional aspects (for an overview, see Tatto et al., 2012).

Approximately 2,000 preservice mathematics teachers in Germany participated in the TEDS-M study in 2008 (among them 1.032 preservice mathematics teachers for primary schools). Following 4 years’ work experience, a subsample of these teachers was re-assessed in the German follow-up study. These teachers gave their consent to be contacted for a follow-up study after they had participated in TEDS-M. After 3 and 4 years, respectively, we contacted all teachers who gave their consent and asked for their willingness to participate in the follow-up study. All data were thus collected on a voluntary basis. As a token of appreciation for their efforts, each teacher was given some didactic material. Given that the teachers who participated in the TEDS-M study were dispersed throughout Germany and to reach as many of them as possible, the TEDS-Follow-Up assessment was developed as an online survey. TEDS-M and its follow-up study were organized in two structurally similar parts: one focusing on primary school mathematics teachers, the other on secondary school teachers. Both parts held equal test components; however, the tests were adapted for the specific school type with respect to content. In this paper, we focus on primary school mathematics teachers and the TEDS-Follow-Up primary instruments.

**Figure 1:**
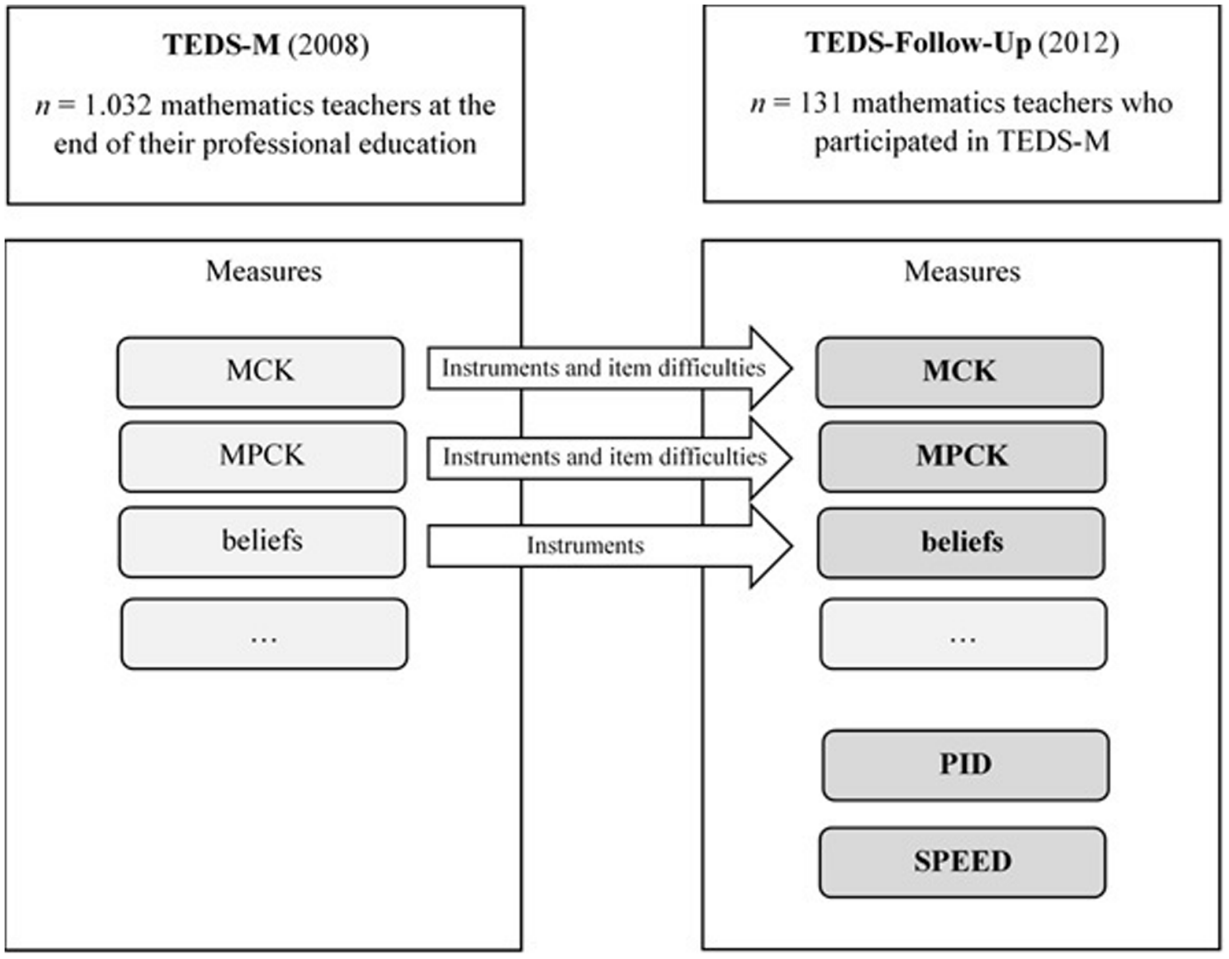
Flow chart on the longitudinal study design of TEDS-M and its follow-up (TEDS-FU).

To assess how the primary teachers’ professional competence developed in the first 4 years of work experience, their MCK and MPCK were re-assessed using parts of the same tests as those used in 2008 as well as adapting the item difficulties from the TEDS-M analyses for data evaluation in 2012 (Follow-Up study). In addition, beliefs and affective-motivational facets were assessed via questionnaire. To get additional insights into situation-specific skills of teachers’ professional competences, a video-based test was newly developed for the Follow-Up study. The test for primary teachers contained three short video vignettes that showed different scenes of mathematics classes with corresponding questions about general, pedagogical, and content-related aspects. Several questions in the video test focused on students’ errors and misconceptions. Therefore, this video-based test is particularly suited to model teachers’ skills of perceiving and interpreting students’ errors and misconceptions as well as deciding how to deal with them (PID skills concerning students’ errors: PID_E). The TEDS-Follow-Up study also included a speed test, measuring teachers’ ability to quickly identify students’ errors in written work (SPEED test). Figure 1 gives an overview of the two studies, their interconnectedness, and measures. For this specific second analysis, we only focus on the data from those test parts that are presented in bold letters and dark grey shading.

### Sample size and characteristics

For this secondary analysis, we used the data from the TEDS-Follow-Up study because it involved crucial test parts to model teachers’ ability to interpret and deal with student errors and misconceptions: the video test as well as the speed test. In the TEDS-Follow-Up study, 231 primary mathematics teachers participated who already took part in the TEDS-M study in 2008. They were approaching the end of their professional teacher education in 2008 and had completed approximately three to four years’ work experience when they were re-assessed in the follow-up study in 2011 and 2012. In the first instance, teachers participated in an online survey in 2011 that included the instruments to assess their beliefs. In the following year (2012), 131 of the 231 teachers participated again in an online test that assessed their professional knowledge [mathematics content knowledge (MCK) and mathematics pedagogical content knowledge (MPCK)], situation-specific skills, and their ability to quickly identify typical student errors.

The 231 primary school mathematics teachers who participated in the online survey in 2011 were mostly female (92%), had an average age of 30 years (min = 27; max = 38), and had achieved an average grade point of 2.5 in their high school exit exams (min = 1.0; max = 3.6). In 2012, the sample characteristics of the 131 teachers showed little variation: average age 30; an average grade point of 2.4 in the high school exit exam; and 90% female.

### Conceptualization of the different test parts

The online assessment comprised several different test parts: (a) a video-based test aiming to assess teachers’ situation-specific skills, (b) a speed test on teachers’ ability to quickly identify typical student errors, and (c) a test on teachers’ mathematics content knowledge (MCK) and their mathematics pedagogical content knowledge (MPCK). Teachers’ beliefs about the teaching and learning of mathematics (d) were assessed a year in advance in the online survey. Each of these test components is described in the sections that follow in addition to the section of the online survey about teachers’ beliefs regarding mathematics teaching.

#### Video-based test assessing teachers’ skills to interpret and deal with student errors and misconceptions

The video-based test, which was developed for the TEDS-Follow-Up study, comprised three short video vignettes with corresponding questions. The three video vignettes varied with respect to their mathematical content (video 1: geometry; video 2: algebraic thinking; video 3: modeling of real-world problems) and with respect to the lesson’s phase (video 1: beginning of the lesson and working phase; video 2: working phase and discussion of results; video 3: beginning of the lesson and working phase). The videos were three to five min long. Prior to each video clip, the teachers read context information about what had happened before the scene started, the mathematical content relevant to the scene, and the students’ backgrounds. This information was accessible as long as necessary and could be re-assessed throughout the entire duration of the test. However, the video was not repeatable in the interest of creating authentic conditions (i.e., actual classes cannot be repeated). After each video, teachers were prompted to answer rating items using Likert scales that asked them to identify relevant aspects as well as open-response tasks relating to the questions concerning teaching situations. The questions that followed the video vignettes focused either on general pedagogical or mathematics pedagogical aspects of the classroom situation. All video vignettes as well as the corresponding questions were intensively validated by expert ratings (see Blömeke et al., 2015b; Hoth et al., 2016b).

Several of these open questions focused on student errors and misconceptions. We selected those questions from the three video vignettes that dealt specifically with students’ errors and misconceptions to model teachers’ abilities in this field. Fifteen items satisfied these requirements. These items focused on analyzing students’ errors and misconceptions from several perspectives: understanding the children’s approaches; thinking about possible causes located either in the lesson itself, the given material, students’ prior knowledge and skills; and anticipating the potential implications of these difficulties for future learning. While the TEDS-Follow-Up video instrument also included items that asked the teachers how they would proceed in the given situation or how they would react to specific incidents shown in the video vignettes, only one item held these decision-making requirements with regard to students’ errors and misconceptions. However, this specific item was excluded from this—newly defined—variable as a result of poor item fit. Therefore, the variable that we newly specify in this secondary analysis focuses on analyzing students’ errors and misconceptions from different perspectives (PID_E).

**Figure 2:**
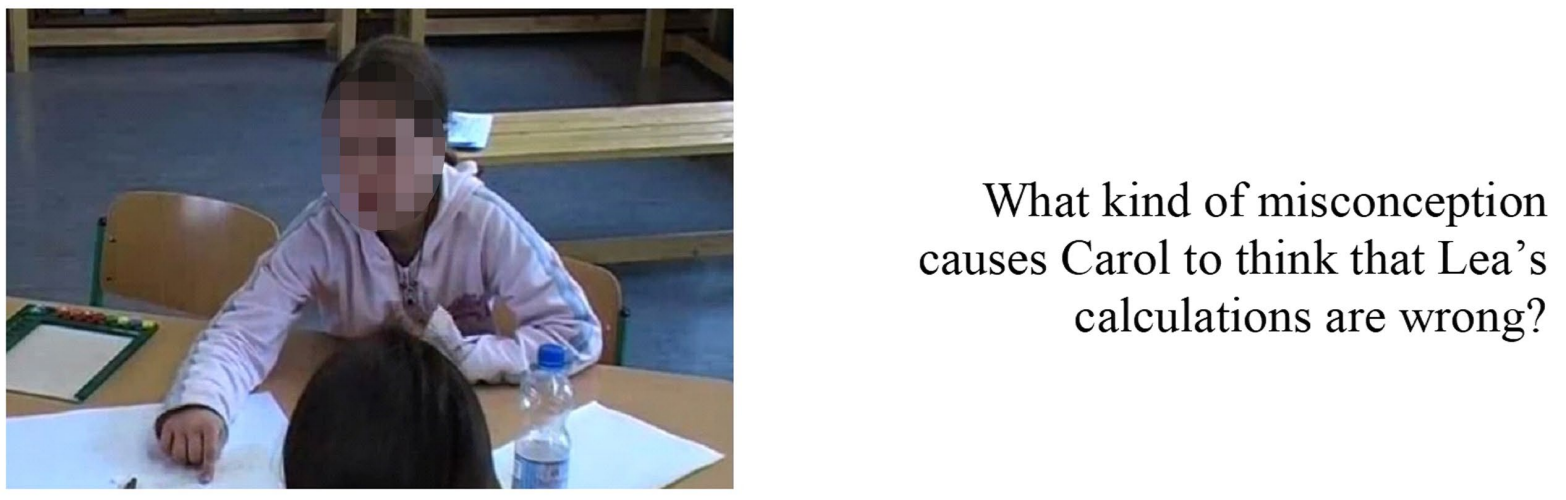
Item example from the video test: analyzing students’ misconception.

Figure 2 shows an example item from the video vignette “real-world problem.” In the video, the teacher presents his third class with a question about sharing payments: Anna and Marc want to go to the movies. Anna pays the tickets for the bus. One ticket costs 2€. Marc pays for the movie tickets. One ticket costs 7€. At the end of the day, the two wishes to compensate their payments. How can they proceed?

**Figure 3:**
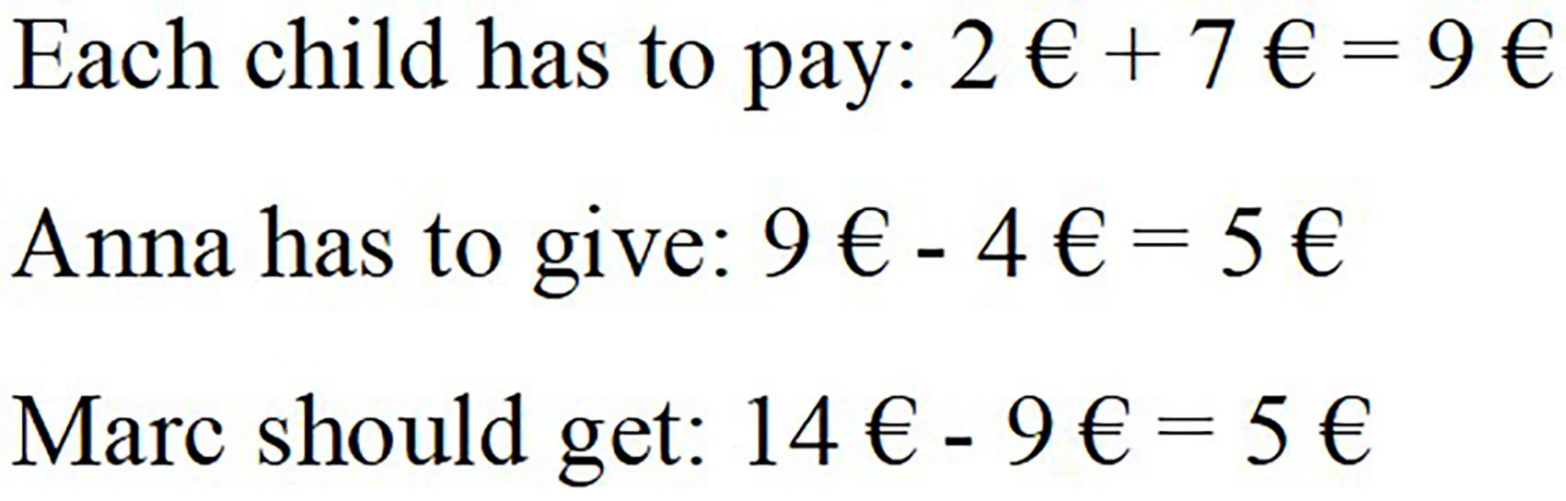
Lea’s solution.

The video depicts several groups of children discussing their approaches. Lea presents her solution (see Figure 3), but Carol is irritated. She says, “But there is a mistake in your calculation. Here, it says ‘gives’ and this line says ‘gets’ but both are subtraction.” Carol perceives the words “get” and “give” as signals for specific operations. For Carol, to get something is always connected to an addition, while giving denotes subtraction. Moreover, Carol does not consider the solution’s underlying context but refers only to the correlation of operation and wording. The question referring to this scene (see Figure 2) asks the teachers to analyze Carol’s misconception.

Teachers’ responses to the 15 items were coded as correct or incorrect on the basis of a coding manual that had been developed in a pilot study with preservice teachers and intensive inductive and deductive coding. For this example task, answers were coded as correct if they indicated that Carol connects those signal words with specific operations and/or does not take the context of Lea’s solution into consideration. One teacher’s example answer that meets these requirements is the following: “Carol is just looking at the signal words “get” and “give” and does not consider the situation’s context.” These binary-scored answers were then further analyzed using IRT methods (see “Data analysis”).

#### Teachers’ ability to identify typical student errors

**Figure 4:**
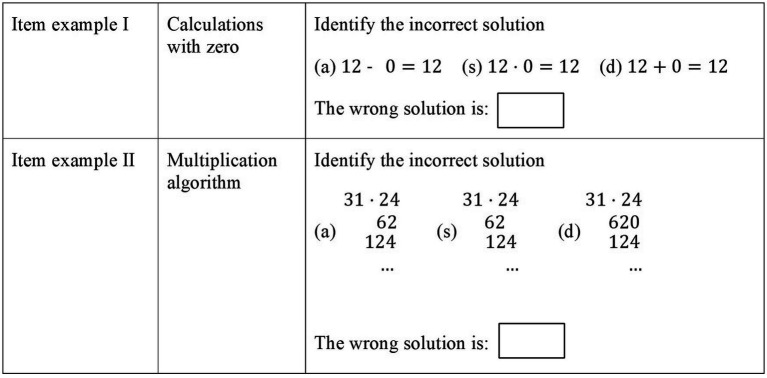
Item examples of the Speed test to quickly identify student errors.

In a second test component, teachers were asked to identify one of three given student solutions that holds a typical error. Here, teachers were given a specific mathematical content or—in some cases—a specific task example and asked to anticipate typical student errors that they might expect for this topic or this specific task. This phase was not time-restricted. Teachers could anticipate potential difficulties for as long as necessary. Subsequently, they were presented with three different students’ solutions. One of these solutions included a typical error, while the others varied in their general approach but were both correct solutions to the task at hand. The teachers were asked to identify the erroneous solution within 4 s. Teachers were unable to test each solution for its correctness within this short timeframe but had to seek specific features in the three solutions that indicated the typical error type. Figure 4 provides two examples. The test comprised 14 items in total and had an acceptable reliability of ${\mathrm{WLE}}_{\mathrm{SPEED}}$ =0.73. For validating purposes, several experts in the field of mathematics education evaluated this test component in a pilot study. In addition, a connecting study validated the speed test of the secondary school test part (e.g., Pankow et al., 2018), which was of equal structure to the one for the primary school test part.

Each of the three student’s solutions was labeled either (a), (s), or (d). Teachers were asked to enter the relevant letter in the box below the three response options. One example item was provided to allow the teachers to become accustomed to this procedure and provide an example of how the three student solutions looked (only minor differences between the three options but one holding a central incorrect approach).

The phase of anticipation of possible student errors plays a very important role, as it activates teachers’ knowledge and allows a quicker identification of the incorrect solution. In example item I, by keeping in mind that calculations with zero can cause specific difficulties for primary school students, teachers can more quickly identify the error in multiplication by zero. In example item II, knowledge of the difficulties of aligning numbers according to their place value in the written multiplication algorithm allows teachers to recognize which approach is incorrect.

#### Mathematics content knowledge and mathematics pedagogical content knowledge

Teachers’ MCK and MPCK were assessed using an adapted version of the TEDS-M tests (for a detailed description of these tests, see Döhrmann et al., 2012 or Tatto et al., 2012). Both test parts included different item formats—single-choice items, multiple-choice items, and open-response questions—and three different cognitive dimensions: knowing, applying, and reasoning (e.g., Tatto et al., 2012). Most items in both test parts were coded as correct or incorrect, while some items were partial-credit items.

During test development for the international TEDS-M study, extensive quality tests were performed to assess validity, reliability, and measurement invariance. For the follow-up study, the MCK and MPCK tests were reduced to shorten the test time for the participating teachers. Given that the TEDS-Follow-Up included additional test parts, such as the video test component, these test parts were reduced in length.

For the MCK test, 25 of the originally 74 MCK items were reused in the follow-up study. All 25 items focused on the content domain of numbers. We focused the reduction on this domain on the basis that it is the predominant topic in German primary mathematics classrooms. This test part showed sufficient reliability ( ${\mathrm{MLE}}_{\mathrm{MCK}}$ = 0.75).

**Figure 5:**
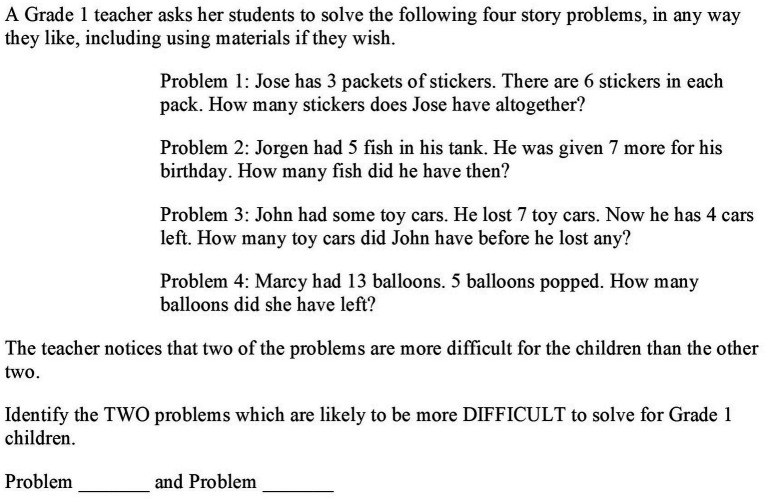
MPCK example item from TEDS-M.

The MPCK test was adopted in its original form from the TEDS-M study. Four items were added from the MPCK test for secondary school teachers to improve the test’s reliability. The contents of those four items were also relevant for mathematics education in primary schools. In total, the test held 37 items that focused on curricular questions, the planning of mathematics lessons, or enacting mathematics for teaching and learning (ibid.). Figure 5 shows an example item. This test part showed good reliability ( ${\mathrm{MLE}}_{\mathrm{MPCK}}$ = 0.79). Since the two tests’ development and conceptualizations were realized in the context of the international TEDS-M study, several experts from different countries were involved in order to create internationally valid instruments. For further information on the test development and validity aspects see Tatto et al. (2012).

#### Teachers’ beliefs about the teaching of mathematics

Teachers’ beliefs were assessed in both studies (TEDS-M as well as TEDS-Follow-Up) using various questionnaires that were informed by valid instruments that were carefully developed and intensely evaluated instruments from international scholars (Tatto et al., 2012). For the specific component of teachers’ beliefs about the teaching and learning of mathematics, a scale with six items was used to gain information about teachers’ constructivist view (Peterson et al., 1989). Teachers rated their agreement to each of the six statements on a 6-point Likert scale (1 = strongly disagree, 6 = strongly agree). High scores on this scale mean that teachers understand mathematical learning as a highly pro-active process that children have to substantially organize for themselves, based on their own inquiry and developing their own approaches to solving problems. Two item examples are the following: (a) “Teachers should motivate their students to find their own approaches to mathematical problems—even if they are inefficient” or (b) “Students are able to find solutions to mathematical tasks without the aid of a teacher.” For each teacher, we determined the average approval to the six statements as a value to indicate their constructivist view. The scale’s reliability was good, with Cronbach’s α = 0.79.

### Data analysis

Multidimensional item response theory methods (Adams et al., 1997; Hartig and Höhler, 2008) were used to (re-)analyze the 131 mathematics teachers’ data. The aim was to find ability parameters for each teacher in the re-organized video test part that merely held a subsample of the original items—only those focusing on student errors and misconceptions. To achieve results for the reduced test that were as reliable as possible, we used the information from the other test parts to analyze the data multidimensionally. The software ConQuest (Wu et al., 1998) was used for the scaling process. For the MCK, MPCK, and the speed-test, scale values already existed from the original scaling processes in the TEDS-Follow-Up study. This scaling process included importing item difficulty parameters from the TEDS-M study for the MCK and MPCK tests, and using the standardization used in TEDS-M, in which person’s ability parameters were set to 500, with the standard deviation set to 100. For the newly scaled test components (PID_E and SPEED), teachers’ ability parameters were first estimated based on the binary raw scores. To simplify interpretations, the mean of teachers’ ability parameters was set to 500 as well, and the standard deviation was set to 100.

Teachers’ ability to analyze student errors and misconceptions during mathematics classes (PID_E) could be scaled sufficiently reliable ( ${\mathrm{WLE}}_{\mathrm{PID}\_E}$ =0.64).

To empirically test the hypothesized relations, the person parameter values for teachers’ knowledge and skills (MCK, MPCK, PID_E, SPEED ability to quickly identify typical student errors) as well as the average consent to the beliefs scale regarding the teaching of mathematics were used in a path model, and relationships were analyzed using the software MPlus 5 (Version 5, Muthén and Muthén, 2011).

## Results

**Figure 6:**
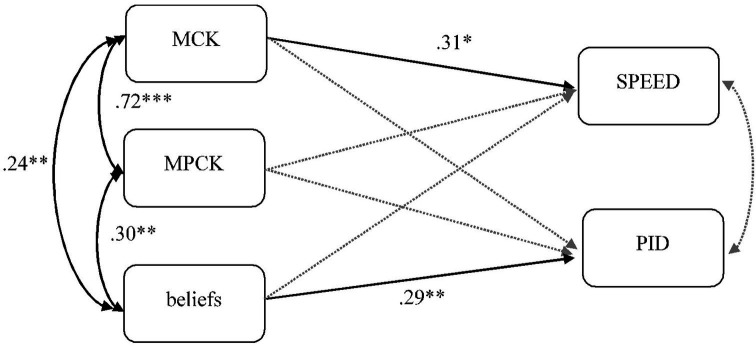
Path model. *p < 0.05; **p < 0.01; ***p < 0.001.

The relevance of content-specific knowledge (MCK and MPCK) to teachers’ ability to analyze student errors in the mathematics classroom was tested in a path model. The newly scaled PID_E variable as well as the SPEED component were used as dependent variables. MCK, MPCK, and beliefs were included in the model as independent variables, as suggested by the competence model developed by Blömeke et al. (2015a) or the professional error competence model developed by Seifried and Wuttke (2010). To identify interdependencies, we analyzed the correlation matrix (Table 1) as well as the predictive power of all three variables in a multiple regression path model (see Figure 6). Table 2 shows the descriptives for all variables, Table 1 presents the correlation matrix between variables, and Figure 6 illustrates the resulting path model.

**Table 1:** Correlation matrix.

	MCK	MPCK	Beliefs	PID_E	SPEED
MCK	1				
MPCK	0.72**	1			
Beliefs	0.24**	0.30**	1		
PID_E	0.30**	0.26*	0.35**	1	
SPEED	0.41**	0.37**	0.12 (n. s.)	0.22*	1

*p < 0.05; **p < 0.01.

**Table 2:** Descriptive results of all analyzed variables.

	Mean	Standard deviation
MCK	533.65	92
MPCK	534.30	85
Beliefs	5.18	0.60
PID_E	500	100
SPEED	500	100

The correlation matrix (see Table 1) shows that teachers’ content-related knowledge (MCK and MPCK) is significantly and positively correlated with teachers’ abilities to identify (SPEED), analyze, and deal with students’ errors (PID_E). This indicates that teachers whose content-related knowledge is high are more frequently able to quickly identify student solutions that incorporate typical misconceptions and more precisely analyze and pick up on these erroneous solutions. Constructivist beliefs regarding mathematics teaching are also positively correlated with teachers’ content-specific knowledge and their ability to analyze and deal with student errors and misconceptions (PID_E). Teachers with constructivist beliefs recognize student errors as a central component in the learning process that may be the origin of learning opportunities. Therefore, they more frequently analyze these mistakes in detail, anticipate possible causes, and contemplate possible approaches to dealing with these errors, while teachers with rather transmissive views may not afford student errors as much room and in-depth consideration.

However, these beliefs are not correlated with teachers’ ability to identify typical student errors quickly (SPEED). This may indicate that the mere identification of typical errors does not automatically lead to reflective approaches in class. This is supported by the fact that teachers’ abilities to (a) identify typical student errors (SPEED) and (b) their ability to analyze and deal with errors in class (PID_E) show relatively low correlation.

However, to analyze how the different variables interact, we define a path model (Figure 6). Here, multiple regression illustrates how the factors interact.

The path model illustrates that in interaction, mathematics content knowledge predominantly predicts how accurately typical student errors are identified in little time (SPEED). By contrast, teachers’ ability to analyze student errors in class (PID_E) is significantly predicted by their beliefs regarding mathematics teaching. The beliefs clearly assume a far stronger role in this relationship than the teachers’ knowledge backgrounds. If teachers believe that mathematics classes should leave sufficient room for the students to find their own approaches to a mathematical phenomenon, they are willing to reframe students’ errors and misconceptions as opportunities for learning.

The residual variances of the SPEED and PID_E facets no longer correlate in this model.

The model depicted in Figure 6 explains 17.4% of the variance in the variable PID_E and 18% variance of the variable SPEED. Clearly, other variables must exist besides teachers’ MCK, MPCK, and beliefs that predict these outcome variables more closely. Other factors may influence the teachers’ situation-specific skills that we did not assess or take up in these analyses.

## Discussion

Models of teachers’ professional competences involve cognitive and affective-motivational aspects that function as dispositional facets. Situation-specific skills (e. g. perception, interpretation, and decision-making skills) are regarded as mediators between a person’s disposition and his or her behavior (Blömeke et al., 2015a). When students’ errors arise during the learning process, dispositional facets, such as knowledge and beliefs, are activated. Teachers only perceive an error when they know that a specific solution is incorrect (referring to content knowledge). Subsequently, teachers must interpret these incidents in light of their knowledge and beliefs and, finally, must decide how they will react (again with close reference to knowledge and beliefs). As Seifried and Wuttke (2010) propose, professional error competence involves (a) specific knowledge about students’ errors and misconceptions, (b) strategies on how to approach errors and failure learning, and (c) constructivist beliefs to understand errors and mistakes as central to learning processes as well as opportunities for future learning.

In this study, we realized a secondary analysis with data from the teachers’ competence study TEDS-Follow-Up to empirically assess the interconnectedness of these different components. Investigating how knowledge and beliefs interrelate with situation-specific skills may yield further insights into how teachers’ competence is formed and how it influences their practices in teaching situations wherein students’ errors arise. We defined two facets as outcome variables for teachers’ competence to effectively deal with students’ errors during class: (a) their ability to quickly identify typical student errors and (b) their ability to analyze student errors and misconceptions in class. Teachers’ ability to quickly identify typical student errors was assessed using a speed test that, after the teachers had been asked to anticipate possible student errors, presented them with three written students’ solutions, of which one was incorrect. We hypothesized that rapid recognition of students’ errors would be relevant for teachers’ competence and would also be related to their professional knowledge. The second facet was assessed using short video vignettes and corresponding questions. To model teachers’ abilities to analyze students’ misconceptions, we selected tasks that specifically dealt with learning from errors and failure and re-analyzed this sub-dataset. We investigated how these abilities interact with teachers’ beliefs and knowledge.

As predicting variables, we used the teachers’ mathematics content knowledge, their mathematics pedagogical content knowledge, and their constructivist beliefs, as assessed in the TEDS-Follow-Up study. The results indicate that a fast identification of typical student errors depends predominantly on teachers’ MCK. However, this ability is not correlated with teachers’ constructivist beliefs. This suggests that some primary school mathematics teachers have robust mathematical knowledge, can quickly identify errors in students’ written work, and espouse more transmissive views on mathematics teaching and learning. It is worth asking, then, what these teachers do upon identifying students’ errors. A teacher-centered and transmissive view of mathematics teaching and learning would be less conducive to deep exploration of student errors, use of them as learning opportunities, and perhaps to teaching all the mathematical content again from the beginning. As existing studies’ findings suggest, such a negative error climate will likely prevent students from learning from their errors (Keith and Frese, 2008; Zhao et al., 2018).

Research has also demonstrated that beliefs exert the strongest impact on teachers’ situation-specific skills. More precisely, they indicate that teachers’ constructivist beliefs are most predictive of their abilities to analyze students’ misconceptions in class. The more teachers understand the teaching and learning of mathematics as a process that is self-constructed by students, the better they will be able to analyze errors and misconceptions in class. The significant impact of teachers’ beliefs is in line with findings from other studies (e.g., Roose et al., 2019; Griful-Freixenet et al., 2020; Keppens et al., 2021). In the multi-regression model, however, teachers’ content-specific knowledge does not explain any additional variance in this ability (PID_E) in addition to the constructivist beliefs. Significant correlation exists between these facets, but beliefs are the most significant predicting factor. As Wilkins (2008) proposes, the effect of content-specific knowledge on situation-specific skills may be mediated by beliefs. These findings shed light on the filtering role that beliefs are thought to play in relation to coping with specific situations. Beliefs seem to determine how teachers interpret classroom situations, what knowledge they activate in this analytical process, and what instructional decisions they make. This is consistent with research evidence suggesting that constructivist beliefs play a strong role in teachers’ competences (Larrain and Kaiser, 2022) and play a role of mediation between teachers’ knowledge and their instructional practices (Leder et al., 2002; Wilkins, 2008; Yang et al., 2020). Moreover, the robust effect of constructivist beliefs on the specific facet of situation-specific skills in error situations is in line with the proposed role that errors play in open and student-oriented teaching approaches. Learners shaping and creating their own learning strategies or exploring non-standard methods more frequently encounter irritation and failure. However, these are understood as valuable opportunities for further learning (Seifried et al., 2022). Teachers who believe that they should encourage their students to explore mathematics and find their own solutions are also more open to listening to students’ explanations and attempting to understand their reasoning. These teachers adopt a flexible approach to understanding students’ thinking, which may trigger in them the need to activate a wider professional knowledge base.

Our study has several limitations. We conducted a secondary analysis of the video test component from the TEDS-Follow-Up study that was developed to assess teachers’ situation-specific skills in different teaching contexts. Given that errors play an important role in mathematics teaching and learning, several questions focused on students’ errors and misconceptions. However, this test was not designed solely to assess teachers’ abilities to identify and interpret errors in classroom situations. Then again, the reliability of this newly scaled facet is only marginally sufficient, suggesting a degree of inaccuracy. Moreover, the TEDS-M tests used to assess teachers’ MCK and MPCK included several tasks from different cognitive and content domains. However, if MCK and MPCK were assessed closely referring to Carol’s specific misconception or the task which is discussed in the video scene, there might be more robust relations between knowledge and situation-specific skills.

Finally, owing to the strong role that constructivist beliefs play in analyzing student errors and misconceptions in class, teachers’ beliefs should play a significantly stronger role in teacher education and professional development. Initial teacher education and professional development programs must go beyond merely providing opportunities to learn content knowledge and pedagogical content knowledge; they must also be concerned with helping teachers and prospective teachers to develop positive and constructive beliefs toward mathematics and its teaching and learning. This may include explicitly addressing teachers’ beliefs about active and student-centered approaches to mathematics learning, as this may determine how (or whether) teachers go on to apply in their classrooms what they have learned at university (or other teacher education setting). More specifically, one main factor that was indicated as relevant to change teachers’ beliefs in favor of constructivism is their reflection on teaching practices (Philipp, 2007; Decker et al., 2015). Therefore, teacher education programs should more closely refer to teachers’ reflections on different scenes of practice in addition to theoretically working through the different learning theories or conceptual change theories. However, teacher beliefs are supposed to be very stable and difficult to change (Philipp, 2007). There is indication that preservice teachers develop more constructivist beliefs within teacher education programs but revert to more transmissive beliefs in the first years of school experience (Voss and Kunter, 2020). If that was true (there are some contradictory findings by Blömeke et al., 2015b), there should be additional support for teachers who start in their teaching practice such as regular reflections on specific scenes from their mathematics classes. Teacher beliefs will shape their analysis of and approach to dealing with errors in the classroom, and so they must be a key element in any teacher education program if the value of their learning content is not to be limited.

Moreover, inclusive school systems and teacher education programs promoting attention to student diversity make more evident the need to pay attention to teachers’ beliefs. Teachers with constructivist beliefs and positive beliefs toward diversity and a differentiated curriculum will more often identify, better interpret, and find more appropriate ways to address students’ errors and difficulties. In addition to professional knowledge and knowledge about inclusive strategies for the primary school classroom, teachers also need to be convinced that they need to pay attention to diversity and that students’ errors can be good opportunities to identifying students’ individual learning needs.

Furthermore, teacher education and professional development programs must provide opportunities for learning how to put dispositions (knowledge, beliefs, etc.) into practice. Noticing courses may provide such opportunities, in that future and in-service primary school teachers receive guiding and support on the selection of events and aspects that are relevant for promoting student learning and on their interpretation based on professional knowledge. These learning opportunities may contribute to build a foundational base to the further development of teachers’ noticing skills during their teaching career.

## Data availability statement

The datasets presented in this article are not readily available because informed consent signed by participants stated that data were only accessible to the authors of this study. Requests to access the datasets should be directed to Gabriele Kaiser, gabriele.kaiser@uni-hamburg.de.

## Ethics statement

Ethical review and approval were not required for the study on human participants in accordance with the local legislation and institutional requirements. The participants provided their written informed consent to participate in this study.

## Author contributions

JH performed the statistical analyses and drafted the manuscript. ML discussed the results of the analyses, contributed to the manuscript, and the revisions. GK provided feedback and ideas and contributed to the revised drafts. All authors contributed to the article and approved the submitted version.

## Funding

TEDS-FU was funded by the German Research Foundation (KA 797/18-1 and BL 548/8–1). The authors are responsible for the content of this publication.

## Conflict of interest

The authors declare that the research was conducted in the absence of any commercial or financial relationships that could be construed as a potential conflict of interest.

## Publisher’s note

All claims expressed in this article are solely those of the authors and do not necessarily represent those of their affiliated organizations, or those of the publisher, the editors and the reviewers. Any product that may be evaluated in this article, or claim that may be made by its manufacturer, is not guaranteed or endorsed by the publisher.
